# Workplace Accidents Among Nepali Male Workers in the Middle East and Malaysia: A Qualitative Study

**DOI:** 10.1007/s10903-018-0801-y

**Published:** 2018-08-02

**Authors:** Pratik Adhikary, Steve Keen, Edwin van Teijlingen

**Affiliations:** 1 Green Tara Nepal, Kathmandu, Nepal; 2 International Care Network, Bournemouth, UK; 3 Faculty of Health & Social Sciences, Bournemouth University, Bournemouth, UK; 4 Manmohan Memorial Institute of Health Sciences, Tribhuvan University, Kathmandu, Nepal; 5 Nobel College, Pokhara University, Kathmandu, Nepal

**Keywords:** Labour, Migration, Asia, Accidents, Interviews, Unintentional injuries

## Abstract

There are many Nepali men working in the Middle East and Malaysia and media reports and anecdotal evidence suggest a high risk of workplace-related accidents and injuries for male Nepali workers. Therefore, this study aims to explore the personal experiences of male Nepali migrants of unintentional injuries at their place of work. In-depth, face-to-face interviews (n = 20) were conducted with male Nepali migrant workers. Study participants were approached at Kathmandu International Airport, hotels and lodges around the airport. Interviews were transcribed and analysed using thematic analysis. Almost half of study participants experienced work-related accident abroad. The participants suggested that the reasons behind this are not only health and safety at work but also poor communication, taking risks by workers themselves, and perceived work pressure. Some participants experienced serious incidents causing life-long disability, extreme and harrowing accounts of injury but received no support from their employer or host countries. Nepali migrant workers would appear to be at a high risk of workplace unintentional injuries owing to a number of interrelated factors poor health and safety at work, pressure of work, risk taking practices, language barriers, and their general work environment. Both the Government of Nepal and host countries need to be better policing existing policies, introduce better legislation where necessary, ensure universal health (insurance) coverage for labour migrants, and improve preventive measures to minimize the number and severity of accidents and injuries among migrant workers.

## Introduction

Nepal is a growing supplier of migrant labour for factories in Malaysia and building sites in the Middle East [3]. Nepal is a low-income country with one quarter of all people living below the nation’s poverty line [8]. Due to limited job opportunities at home, international migration is considered as a livelihood strategy for many Nepali men with most working in high-risk, unskilled and low-paid jobs. It is estimated that about 4 million Nepalese are working abroad as temporary workers or so-called ‘guest workers’; primarily in Malaysia, the Middle East and India. These migrant workers send home over US $4 billion back home every year, comprising over 30% of Nepal’s gross domestic product [12].

The theoretical underpinning is the dual labour market theory initially proposed by Piore [27]. This theory associates labour migration specifically to the host economy as it explains migration from the demand side [23]. Labour migrants from less developed economies travel to fill the unskilled and low-skill jobs as guest workers in more developed economies to do the jobs better trained and paid local workers do not want to do. This theory also explains the active recruitment through labour agents in Nepal to help fulfil the demand for labour abroad, and it helps explain some of the exploitation highlighted in host countries. People working in another country (i.e., migrant workers) do so at a higher risk to their health than native workers [1, 4, 20]. Similar to migrant workers from around the world, Nepali migrant workers also experience serious health and safety problems in the host countries including accidents and injuries. A recent study on Nepali migrants suggested that there are more than 1000 deaths annually in the Middle East and Malaysia [7]; 10% are due to suicide. Previous studies with Nepali migrant workers returning to Nepal have established that they often work in risky occupations and frequently face accidents and injuries [2, 3, 21]. The latest review aiming to identify gaps in Nepali migrant workers’ health and well-being found that more than half (11 out of 18) of the articles focused on sexual health and/or HIV, with considerably fewer focused on work-related risk factors and lifestyle factors in migrant workers [33]. This in-depth qualitative study explores the nature of accidents and injuries of male Nepali workers abroad. The study is meant to highlight migrant workers’ experiences of work-related accidents and to identify factors that participants perceived as related to these accidents.

## Methods

This study is based on qualitative, in-depth interviews [13] to explore workplace accidents of Nepali male migrant construction and factory workers. Since there is no formal data base of migrant workers in Nepal participants were approached at the country’s only international airport, Tribhuvan International Airport, in the capital Kathmandu. Unless people travel to or through India the overwhelming majority of migrant workers passes through Tribhuvan International Airport. Migrant workers were approached upon their arrival or before departure, recruited for the study and subsequently interviewed. Due to the limited time people spent at the airport, arriving migrant workers who were interested in participating were asked to meet later and interviewed at temporary residences (e.g., guest houses/hotels/lodges) in Kathmandu where they often spent a few days. After two pilot studies [35], 20 participants agreed to be interviewed to help us gain a deeper understanding of the working conditions in their host country. Consent to participate was obtained from each person prior to the interview. Data collection took place between July and September 2011. Semi-structured interviews [14] were conducted in Nepali by the first author who is a native speaker. Purposive sampling was used [25, 26] to include a range of migrant workers from different age groups, working in different countries (Qatar, Saudi Arabia and Malaysia), with or without experience of accidents, and with or without good self-reported work environment. As the experience of accidents was of critical interest, participants were purposively selected so that almost half of the participants had experienced some sort of accident (e.g., cuts, falls, fractures and other injuries) at work. To help interpret the participants’ perceptions and experiences we also asked them a range of demographic factors. Table 1 includes ‘country’ where they had worked abroad, their age, highest level of education achieved, marital status and caste/ethnic group. Combining the latter is the convention in official documents in Nepal, the 2011 Census reported that there are “125 caste and ethnic groups” in the country [10]. In addition we added the categories ‘health’ which is their self-reported health status and ‘work’ their self-reported working conditions, both using the categories: ‘very poor’, ‘poor’, ‘fair’, ‘good’ and ‘very good’, and the length of stay reporting the number of years they had worked abroad. The overwhelming majority of the population of Nepal is Hindu [34] and in the caste system Brahmin/Chhetri represent the most advantaged group, Dalits are the most disadvantaged group and other ethnic groups mentioned are situated somewhere in between in the caste system.

**Table 1:** Socio-demographic characteristics of participants

Countries	Ages	Accidents	Health	Work	Caste/ethnic groups^a^	Education^b^	Married	Length of stay (years)
Qatar	28	No	Fair	Poor	B/C	Intermed.	Yes	2
Saudi Arabia	29	No	Good	Fair	B/C	Primary	Yes	10
Malaysia	23	No	Good	Good	Dalit	SLC	Yes	4
Saudi Arabia	48	Yes	Fair	Fair	Dalit	None	Yes	8
Saudi Arabia	49	Yes	Poor	Good	Cha	SLC	Yes	13
Malaysia	42	Yes	Fair	Fair	Limbu	SLC	Yes	2
Qatar	41	Yes	Poor	Poor	Tamang	None	Yes	6
Malaysia	20	No	Poor	Poor	B/C	SLC	Yes	1
Malaysia	25	No	Poor	Poor	B/C	Primary	Yes	3
Saudi Arabia	40	Yes	Very poor	Poor	B/C	None	Yes	5
Qatar	23	No	Good	Good	B/C	SLC	No	4
Saudi Arabia	21	Yes	Very poor	Poor	Limbu	Primary	No	2
Malaysia	35	Yes	Very poor	Poor	B/C	SLC	Yes	1
Malaysia	25	No	Good	Fair	Dalit	Primary	No	6
Malaysia	38	No	Poor	Fair	Tamang	Primary	Yes	2
Qatar	40	No	Poor	Poor	Dalit	Primary	Yes	6
Qatar	29	No	Good	Good	B/C	Primary	Yes	3
Qatar	27	No	Good	Good	B/C	SLC	Yes	2
Qatar	22	Yes	Good	Good	Magar	SLC	No	2
Qatar	21	Yes	Good	Good	B/C	SLC	No	2

Work = working condition

^a^Ethnicity: B/Cs = Brahmin and Chhetri, Cha = Chaudhary

^b^Education: Primary = primary school, SLC = school leaving certificate, Intermed. = intermediate level

In this qualitative study, interviews were recorded on a digital audio recorder [13]. Each interview lasted on average about 45 min. Data were transcribed verbatim in Nepali and then translated by the first author (PA). All three authors read and coded all transcripts independently. A second bi-lingual Nepali with a research background in Public Health translated back into Nepali a portion (20%) of the transcripts originally translated into English by the first author. This process of quality control of the translation process is known as back-translation [31]. This second translator came up with almost identical words which gave assurance of the quality of the first author’s original translation.

The qualitative data were analysed using a thematic analysis [9]. Applying an inductive coding process [18, p. 252], any discrepancies and difference in codes were discussed by all three authors together and final themes were agreed. Ethical approval was obtained from the Ethics Committee of the Nepal Health Research Council (no: 462 + 1190).

## Findings

Table 1 presents the socio-demographic and work-related details of the 20 participants.

Their ages ranged from 20 to 49 years, with the majority (60%) less than 30 years old, and three-quarters (n = 15) were married. Half of the participants were from privileged (= higher) caste groups (Brahman/Chhetri); whilst four were from the most disadvantaged group (Dalit) (Table 1). Half of these migrant workers either had no education or had only completed primary education. Two-thirds of participants worked in the Middle East and one-third worked in Malaysia. More than half (55%) rated their health as fair or good. The majority (60%) rated their work environment as fair or good. Nearly half had experienced work-related accidents. Of those who experienced work-related accidents, two worked in Malaysia and the remainder in the Middle East.

### Theme and Categories

During data analysis, seven themes arose related to how participants experienced accidents and injuries abroad. These themes are: (1) risk-taking practices (at work and not at work), (2) perceived work pressure, (3) employer type, (4) health and safety, (5) temperature at work, (6) working hours, and (7) communication issues. These themes are inter-related and fit one overarching theme about work-related accidents among migrant workers ‘unsafe work environments’.

Some migrant workers spoke about their own experience of workplace accidents and injuries and others recounted those of friends or colleagues. The work-related accidents described ranged from minor accidents with no long lasting impact to serious incidents causing life-long disability. Not all accidents happened due to structural factors such as working conditions and some we perceived as due to workers’ taking risks themselves.

### Risk-Taking Practices: (a) At Work

During their employment abroad, migrant workers perceived they were at a higher risk of accidents and injuries. Sometimes migrants have to make difficult decisions to help co-workers without appropriate protective mechanisms. A young construction worker who had experienced a very serious accident recalled:

One day one of my colleagues asked for help to fit a 2000 ton (NB the interviewee probably meant to say: 2000 kg) machine on the top of a building. Actually that was not part of my job but I agreed to help him. Then we tried to put the machine on the stand using a forklift although normally workers used a crane for such work. The machine however did not fit properly on the stand and the machine fell down and crushed half of my body.

(Limbu, Low edu. Poor health, Middle East, Age 21)

He was no longer able to work either abroad or in Nepal as he is now seriously disabled. When he was asked about the impact of the accident he explained that the accident had ruined his life:

I am unable to walk, can’t go to meet friends. I have to struggle even to go to the toilet, take a shower or go outside. I need help for this. I am disturbed mentally. I am single and question myself how can I survive. I am thinking of asking the government for facilities as a disabled person.

(Limbu, Low edu. Poor health, Middle East, Age 21)

### Risk-Taking Practices: (b) Not at Work

Several participants shared that Nepali migrant workers often drink alcohol after their shift (in the evening). They added that after drinking alcohol, a worker might lose his decision making ability. A factory worker in Malaysia gave an example of an alcohol-related traffic accident.

One of my friends went to the market nearby our accommodation. He was drunk while returning to his accommodation. It was about at 9 pm. He was in a group of four or five friends. He did not take care about the traffic light. He crossed the road on a red traffic light and that caused an accident. It was his mistake:

(Dalit, SLC, Good health, Malaysia, Age 23)

### Perceived Work Pressure

In addition to engaging in risky work practices, migrant workers were also at risk of accidents and injuries owing to pressure from senior members of staff. One of the study participants working in a factory in Malaysia said, pointing to his right hand, “I lost my four fingers”, and explained in some detail:

I worked in a biscuit factory. My supervisor was Chinese and he put pressure on me at work. I didn’t understand his language. During preparation of cream to make the biscuits I was trying to put sugar in the mixture. I always stopped the machine while putting items in it but that day my supervisor told me to put it in while the machine was still running. He was standing at my side. I poured the sugar in the running mixture, and it cut four of my fingers.

(Limbu, High edu. Fair health, Malaysia, Age 42)

A construction worker in Saudi Arabia reported:

There is a strict work environment. The employer puts a great deal of pressure on us. The manager or owner has threatened us that they will reduce our salary if we are unable to complete a task within a fixed time.

(Dalit, No education, Fair health, Saudi Arabia, Age 48)

### Employer Type

A number of participants explained that occupational risk is related the type of employer or company they worked for. The bigger companies appeared to be better employers than the smaller ones in terms of health and safety. A construction worker in Qatar said:

There were more accidents in the small companies compared to the big companies. The small companies had less health and safety at work and people experienced more accidents.

(Tamang, No education, Poor health, Middle East, Age 41)

A factory worker based in Malaysia also highlighted that:

Accidents and injuries depend on the company or factory. People didn’t have accidents and injuries in the good companies or in workplaces where they do not have to work with machines. People who worked using heavy machines have more chances of having accidents and injuries. People using smaller machines were less likely to get accidents and injuries.

(B/C, SLC, Poor health, Malaysia, Age 20)

### Health and Safety

Health and safety is a prime concern of most workers. Many migrants perceived they worked without appropriate health and safety regulations at work and that this increased their risk for of falls, cuts, and other accidents as a result of, for example, poorly maintained equipment. A number of workers who had fallen from the tops of buildings became disabled.

A participant who had experienced an accident recalled:

One day I was working on a hole to pass the sewage pipe through the wall to the fourth floor. I was not wearing a safety belt or helmet that day. I slipped and fell from the fourth floor and was trapped in the hole. My back bone, legs and hands broke. I also have a vision problem.

(B/C, No edu. Poor health, Middle East, Age 40)

When he was asked about his feelings during and after the accident he reflected:

I thought I was at the final stage of dying. I didn’t have any hope that I would live. I was really worried whenever my friends visited me. Later I felt a little better although I had no hope for my life and the future. Sometimes, I thought it would have been better if I died rather than staying in this situation.

(B/C, No edu. Poor health, Middle East, Age 40)

After the accident, he felt bitter that he had been placed in the worst possible situation economically. He argued:

I have borrowed NRs 300,000 ($3281) to go abroad. I don’t have sufficient funds or property to repay. I feel sad. I have two sons aged 13 and 11. I cannot imagine how my children will pay back that money.

(B/C, No edu. Poor health, Middle East, Age 40)

A migrant worker in Saudi said that Nepali migrants faced accident when they work on scaffolding without applying health and safety measures. He added:

One Nepali who was a lead man in our company had an accident abroad. The crane lifted up a block. The crane driver was on the top and our Nepali friend was at the bottom. The rope of the crane broke and the block fell and hit our Nepali friend on the head. His hands, legs and head were injured.

(Chaudhary, High edu. Poor health, Middle East, Age 49)

### Temperature at Work

Most of the construction workers in this study experienced very high temperatures at work because they were based in the Middle East and worked outside. A construction worker in Saudi Arabia argued:

The temperature was too hot and we worked all day without water. Workers fainted due to unbalance of hot and cold temperature when they drank cold water in the hot work environment.

(B/C, No edu. Poor health, Middle East, Age 40)

Similarly, another worker from Qatar added:

It is difficult to work in the hot environment which leads to sweating, dehydration and heat stroke, but I have not faced any major problems.

(B/C, SLC, Good health, Middle East, Age 23)

One of the construction workers articulated:

The work environment was very hot. We sweated all the time because of high temperatures. Sometimes we wanted to leave the job and return to Nepal.

(Chaudhary, High edu. Poor health, Middle East, Age 49)

In contrast, factory workers in Malaysia were more positive about temperatures at work because they were based in Malaysia and worked inside. One of them shared his view:

The work environment in my company was not too bad. The company provided a fan. So, the environment was okay.

(Tamang, Low edu. Poor health, Malaysia, Age 38)

### Working Hours

Several study participants in both Middle Eastern countries mentioned that they worked long hours. One of the construction workers in Qatar said:

We worked from 5 am in the morning to 5 pm in the evening. We only got our lunch at 2 pm in the afternoon. All the other time, we only drank water and worked without any snacks.

(Tamang, No edu. Poor health, Middle East, Age 41)

In contrast many factory workers in this study explained that they had an option of working short shifts. One of them said:

It is difficult to work 12 hours shifts. I preferred to work short hours, i.e. 8 hours per day. A short shift is less boring. That’s why I was happy to work.

(Dalit, High edu. Good health, Middle East, Age 23)

### Communication

Many participants noted that upon their arrival Nepali workers had limited knowledge of the nature of their job and poor communication skills with colleagues and senior staff. Workers were neither confident in English nor understood the language of the host country. A number of study participants believed that communication difficulties with colleagues and supervisors might have increased the risk of accidents and injuries at the workplace. One of the factory workers explained:

Workers also face accidents because of language problems. There are many supervisors and managers from different countries, for example, from China. So, it is difficult to understand their language and people work differently than is recommended, thereby increasing the risk of accidents at the work place.

(B/C, High edu. Poor health, Malaysia, Age 20)

A migrant worker in the Middle East specifically mentioned language and translation problems:

The main problem is communication between workers to workers and senior to junior workers; although, the employers provide information about health and safety at work, workable temperature and so on, Nepali workers do not understand the language of the host country.

(B/C, High edu. Fair health, Middle East, Age 28)

A migrant worker in Malaysia said something similar as he implied that Nepali migrant workers worked largely without clear instructions and communication as they did not understand the local language:

The work environment was not good. We didn’t understand the language. We only worked.

(B/C, High edu. Poor health, Malaysia, Age 35)

## Discussion

The aim of the study was to explore the personal experiences of Nepali migrants around workplace related unintentional injuries. The results of the study point out that some migrant workers experienced a wide range of accidents and accidents, including more serious injuries. Risk-taking among workers during their work increased workplace accidents and injuries. One example of risk taking is that a migrant worker was willing to help his friend (although it was not part his job) to fit a machine on a stand without using correct safety procedures. As a result the machine fell down and crushed half of his body. Such horrific accidents were also noted in an exploratory study among immigrants in US [6]. Guldenmund et al. [19] also confirmed the findings of this study concluding that migrant workers cannot ‘afford’ to be to concerned in their own safety and health as they moved abroad to earn money. Consistent with this study, previous descriptive studies of Nepali migrants working in the Middle East and Malaysia also highlighted that these workers experienced accidents and injuries abroad [3, 21].

In addition to engaging in risky work practices, migrant workers are also at risk of getting accidents and injuries due to the pressure of senior member of staff or supervisor, perhaps resulting in not using personal protection equipment (PPE) or not using it adequately. One of our study participants working in a factory in Malaysia lost four fingers due to his supervisor ordering him to work on a dangerous machine. Whilst, a migrant construction worker in Singapore reported that doing tasks under pressure of time increases the risks of accidents and injury. Kontos et al. [22] and Dutta [17] also found that workers in their study experienced pressure to work unsafely.

Migrant workers who participated in an in-depth interview believed that their risk of getting accidents was due to inadequate protection, including PPE, at work or because of poor working conditions. For example, a number of workers had fallen from the tops of buildings and become disabled or died. The kind of reasons for having accidents raised by participants are consistent with the results of earlier studies [5, 30]. Associations between working conditions (i.e., long working hours, language problems, lack of safety training) and health problems including accidents at work are well documented in migrants working in unskilled jobs [11, 15, 17, 24, 36, 37].

The risk of acquiring accidents among workers increased as a result of working in high temperatures. This study identified that migrants working outdoors in the Middle East experienced excessive heat at work leading to sweating, dehydration and heat stroke. In contrast, factory workers were more positive about temperatures at work because they were based in Malaysia and worked inside. Research confirms that working in very high temperatures is a risk factor for accidents and injuries [3, 21, 29]. The quote used above to illustrate the high temperatures in the Middle East “Workers fainted due to unbalance of hot and cold … when they drank cold water in the hot work environment” relates traditional beliefs in humeral medicine. This notion has been raised in a different setting by Quandt et al. [28] in a study of USA farmworkers who believed that some are inherently more susceptibility to the health effects of chemical exposure than others. Sharma et al. [32] report on similar traditional beliefs about pregnancy and childbirth in Nepal, including notion around hot and cold foods which do not relate to temperature or its spiciness.

This study has also found that occupational risk is related to kind of employer or company they work for. The bigger companies are safer to work for than the smaller ones in terms of insurance coverage and health and safety at work. Migrants employed in small companies are therefore more likely to experience accidents at work. Similarly, the results of Arcury et al. [6] on occupational safety beliefs among Latino residential roofing workers suggested that health and safety of workers is related to company size and number of employees.

In the present study, part of the explanation for migrants experiencing accidents at work abroad appears to be due to communication difficulties with colleagues and supervisors who are often from different countries. Dutta [17] highlighted that Bangladeshi migrant construction workers in Singapore had accidents due to poor communication; a similar issue was found in our study. Nepali migrant workers at the bottom half of the dual-labour market economy, generally have low education levels, speak little or no English, nor are they likely to speak local languages. Supervisors and managers are often other foreign workers who speak no Nepali, and perhaps also poor English.

Some of the issues raised in this paper also link closely the notion of temporary migrants [16]. Temporary migrants’ stays in the host country is seen as time-limited, this seems to be especially true for Nepali migrant workers to the Middle East who are often on contracts for 2 or 3 years, they travel alone to the host country leaving partners and children behind in Nepal. However, many of these issues link closely to the dual labour market theory. Nepali migrant workers in both Malaysia and the Middle East end up doing the work that local workers no longer want to do as they have access to a labour market of better paid jobs.

Strengths and weaknesses of the study: this is one of the first qualitative studies of its kind based on Nepali migrant workers, benefiting for the language skills of the first author, a native Nepali speaker. Weaknesses include the delay in reporting. The research is part of the first author’s PhD study (awarded in 2016), although: (a) a recent overview of research into Nepali migrant workers [33, p. 1], reported that more that most “articles discussed sexual risk taking and HIV, whilst considerably fewer focused on work-related risk factors and lifestyle factors”, and (b) recent international media coverage of migrant workers in the Middle East suggests that their working conditions have certainly not improved. Also not being able to do the study in host countries meant migrant workers needed to be captured at the main international airport in Kathmandu this will have resulted in an element of self-selection.

## Conclusion

This qualitative study has identified seven related sub-themes about the work and working conditions of Nepali migrant workers: risk-taking practices, perceived pressure from employer, type of employer, health and safety issues, working hours, communication issues and physical working environment especially temperature. Some sub-themes relate more to the individual worker(s), others more to the structural work environment, some relate to both. It is important to separate such issues as some can be address by better training whilst other require change in laws or monitoring of legislation around health and safety, and again others require trade union action or international public pressure on exploiting employers. Communication problems could be reduced through structural solutions such as firms appointing Nepali-speaking supervisors, but also individual solutions such as migrant workers learning sufficient English, Chinese, Hindi or Malay (depending on the job they are applying for).
